# Memory Impairment and Plasma BDNF Correlates of the *BDNF Val66Met* Polymorphism in Patients With Bipolar II Disorder

**DOI:** 10.3389/fgene.2018.00583

**Published:** 2018-11-27

**Authors:** Yun-Hsuan Chang, Tzu-Yun Wang, Sheng-Yu Lee, Shiou-Lan Chen, Chih-Chun Huang, Po See Chen, Yen Kuang Yang, Jau-Shyong Hong, Ru-Band Lu

**Affiliations:** ^1^Department of Psychology, College of Medical and Health Science, Asia University, Taichung, Taiwan; ^2^Department of Medical Research, China Medical University Hospital, China Medical University, Taichung, Taiwan; ^3^Department of Psychiatry, National Cheng Kung University Hospital, College of Medicine, National Cheng Kung University, Tainan, Taiwan; ^4^Dou-Liou Branch, Department of Psychiatry, National Cheng Kung University Hospital, Yunlin, Taiwan; ^5^Department of Psychiatry, Kaohsiung Veteran’s General Hospital, Kaohsiung, Taiwan; ^6^Department of Psychiatry, College of Medicine, National Yang-Ming University, Taipei, Taiwan; ^7^Department of Psychiatry, Faculty of Medicine, Kaohsiung Medical University, Kaohsiung, Taiwan; ^8^M.Sc. Program in Tropical Medicine, College of Medicine, Graduate Institute of Medicine, Kaohsiung Medical University, Kaohsiung, Taiwan; ^9^Department of Medical Research, Kaohsiung Medical University Hospital, Kaohsiung, Taiwan; ^10^Institute of Behavioral Medicine, College of Medicine, National Cheng Kung University, Tainan, Taiwan; ^11^Neurobiology Laboratory, National Institutes of Health/National Institute of Environmental Health Sciences, Research Triangle Park, NC, United States; ^12^Beijing YiNing Hospital, Beijing, China

**Keywords:** bipolar II disorder, BDNF genotype, plasma concentrations of BDNF, memory, auditory delayed memory

## Abstract

Studies suggest that a functional polymorphism of brain-derived neurotrophic factor (BDNF), polymorphism *BDNF Val^66^Met* affects cognitive functions, however, the effect is unclear in bipolar II (BD-II) disorder. We used the Wechsler Memory Scale-third edition (WMS-III), the presence of the *BDNF Val^66^Met* polymorphism, and plasma concentrations of BDNF to investigate the association between memory impairment and BDNF in BD-II disorder. We assessed the memory functions of 228 BD-II patients and 135 healthy controls (HCs). BD-II patients had significantly lower scores on five of the eight WMS-III subscales. In addition to education, the BDNF polymorphism were associated with the following subscales of WMS-III, auditory delayed memory, auditory delayed recognition memory and general memory scores in BD-II patients, but not in HC. Moreover, BD-II patients with the *Val*-homozygote scored significantly higher on the visual immediate memory subscale than did those with the *Met/Met* and *Val/Met* polymorphisms. The significantly positive effect of the *Val*-homozygote did not have a significantly positive effect on memory in the HC group, however. We found no significant association between BDNF polymorphisms and plasma concentrations of BDNF. The plasma BDNF was more likely to be associated with clinical characteristics than it was with memory indices in the BD-II group. The impaired memory function in BD-II patients might be dependent upon the association between the *BDNF Val^66^Met* polymorphism and peripheral BDNF levels.

## Introduction

Patients with bipolar disorder (BD) have a variety of cognitive deficits (Bearden et al., 2001; Murphy and Sahakian, 2001; Quraishi and Frangou, 2002) that might affect drug adherence, therapeutic outcomes, and prognosis (Simonsen et al., 2008). Most prior studies focused on patients with bipolar I disorder (BD-I) (Dickerson F. et al., 2004; Dickerson F.B. et al., 2004; Jamrozinski et al., 2009). Bipolar II disorder (BD-II) patients have a more chronic course with depressive episodes and shorter periods in remission than do BD-I patients (Judd et al., 2003). Rihmer and Kiss (2002) found that BD-II patients had the highest risk of suicide, interpersonal conflict, and family breakdown among people with major mood disorders. In addition, there is no consensus on whether BD-I and BD-II are different subtypes of BD. Evidence from biological, clinical, and pharmacological studies indicates that they are (Vieta et al., 1997; Vieta and Suppes, 2008; Wang et al., 2012). Moreover, BD-I and BD-II patients are reported to have two distinct genotypes (Charney et al., 2017). Compared with healthy controls, BD patients had significantly poorer executive function and memory that was more impaired than did healthy controls during remission (Martinez-Aran et al., 2004). This high correlation between poor memory and learning might explain the daily dysfunction in BD patients during remission.

Evidence has shown a negative effect of mood episode duration and severity on memory performance and executive function (Cavanagh et al., 2002; Martinez-Aran et al., 2004; Bearden et al., 2006). The more mood episodes the patients had, the poorer their higher order cognitive performance would be (Ciammola et al., 2007; Levy et al., 2009). Gualtieri and Johnson (2006) reported that verbal memory impairment seemed to present across mood phases, stable marker which implied that it was a stable marker for BD.

Brain-derived neurotrophic factor (BDNF), a member of the neurotrophins family, is important for neuron survival and proliferation (Berk et al., 2011). More specifically, BDNF is highly correlated with learning- and memory-related hippocampal neurons (Poo, 2001; Lu et al., 2008). Guzowski et al. (2000) reported that inhibiting BDNF signaling by gene knockdown or antisense RNA would impair spatial learning and memory. Altar et al. (1997) reported an association between BDNF and dopamine survival and functioning in the midbrain. Preclinical evidence suggests BDNF is involved in the hippocampal functions of learning and memory (Hariri et al., 2003). An SNP (rs6265) producing a valine (*Val*)-to-methionine (*Met*) substitution in the pro-BDNF protein at codon 66 (*Val^66^Met*) correlates with hippocampal-mediated memory performance in humans (Egan et al., 2003; Hariri et al., 2003). In addition, Toh et al. (2018) recently reported that *Val*-homozygotes had better performance in the tasks related to memory in healthy participants and clinical population.

Kim et al. (2010) reported a significant postmortem decrease of serum BDNF level in BD-II patients, which indicates an association with brain atrophy and progressive cognitive changes in BD-II. Pan et al. (1998) concluded that there is a positive correlation between serum and central BDNF levels. Serum BDNF levels gradually decline in the later stages of BD episodes (Kapczinski et al., 2008a; Kauer-Sant’Anna et al., 2009). Studies have also reported lower serum BDNF levels during manic and depressive episodes, and higher levels during remission (Kapczinski et al., 2008b; Lin, 2009).

BDNF might be a biomarker of normal cognitive function in healthy adults (Yu et al., 2008). In addition, BD patients with *Val* homozygotes are more likely to perform better cognition (Mandolini et al., 2018). Moreover, serum BDNF levels fall significantly in progressive cognitive decline, e.g., mild cognitive impairment (Yu et al., 2008), Alzheimer’s disease (Gunstad et al., 2008), Huntington’s disease (Ciammola et al., 2007), and schizophrenia (Zhang et al., 2012). Furthermore, peripheral BDNF levels have therapeutic effects on acute mania episodes, but not on depressive episodes (Fernandes et al., 2015). However, Chen et al. (2014) concluded that “plasma BDNF profiles in different mental disorders are not affected by *BDNF Val^66^Met* gene variants, but by the process and progression of the illness itself,” and they suggested a positive correlation between BDNF levels in the peripheral and central areas. Thus, the plasma concentration of BDNF can be examined as a possible biomarker for the clinical course of BD.

We hypothesized that the BD-II group would have relatively lower scores than would the HC group in some memory performance domains. In addition, such memory impairment is correlated with various BDNF polymorphisms. Moreover, the plasma concentration of BDNF might be a biomarker for predicting memory performance in BD-II patients.

## Materials and Methods

### Participants

The research protocol was approved by the Institutional Review Board (IRB) for the Protection of Human Subjects at National Cheng Kung University Hospital. All the participants were given full information about the study and provided a signed written informed consent form. The patient groups were recruited from outpatient and inpatient settings at National Cheng Kung University Hospital. Each patient was initially evaluated by an attending psychiatrist and then interviewed by well-trained research team members using the structured interview of the Chinese Version of the Modified Schedule of Affective Disorder and Schizophrenia-Life Time (SADS-L) (Endicott and Spitzer, 1978), which has good interrater reliability (Huang et al., 2004) and is based on the diagnosis of BD in the *Diagnostic and Statistical Manual of Mental Disorders, fourth edition-TR* (DSM-IV-TR). Patients diagnosed with any illness other than BD-II were excluded. The Young Mania Rating Scale (YMRS) (Young et al., 1978) and the Hamilton Depression Rating Scale (HDRS) (Hamilton, 1960, 1967) were used to evaluate the severity of their mood symptoms. Healthy controls were volunteers recruited from the community via advertisements. The Chinese version of the SADS-L was used to screen and exclude all with a history of psychiatric illness. All HCs were free of present or past major mental illness (affective disorder, schizophrenia, anxiety disorder, personality disorder, and substance use disorders), and none had a family history of psychiatric disorder among their first-degree relatives. Moreover, only Han Chinese participants were recruited to reduce the effect of ethnic heterogeneity in the genetic analysis.

### Measuring Plasma Concentrations of BDNF

Twenty milliliters of blood was drawn using venipuncture from each participant. Plasma was isolated from the whole blood after it had been centrifuged at 3000 *g* for 15 min at 4°C, and then it was immediately stored at -80°C. The BDNF levels were quantified using an antibody pair assay system (Flexia; BioSource Intl., Camarillo, CA, United States). Sample processing and data analysis were done according to the manufacturer’s instructions. The genotypes of the *BDNF Val^66^Met* polymorphisms were determined using polymerase chain reactions plus restriction fragment length polymorphism (PCR-RFLP) analysis (Neves-Pereira et al., 2002). The laboratory technician who did the genotyping and read out the genotype data was blinded to the patients’ diagnoses. The genotype error rate was less than 5%.

### Wechsler Memory Scale-Third Edition

The Wechsler Memory Scale-third edition (WMS-III) (Wechsler and Stone, 1997) is the most commonly used test for memory functions. Composite scores were calculated for the eight standardized primary indices: Auditory Immediate Memory Index (WMS-AIM), Visual Immediate Memory (WMS-VIM), Immediate Memory (WMS-IM), Auditory Delayed Memory (WMS-ADM), Visual Delayed Memory (WMS-VDM), Auditory Delayed Recognition Memory (WMS-ADRM), General Memory (WMS-GM), and Working Memory (WMS-WM).

### Statistics

Pearson χ^2^ analysis was used to examine the gender differences, BDNF genotype distribution between groups, and other categorical variables. The distribution of alleles and genotypes were calculated and compared between groups using χ^2^ tests. Hardy-Weinberg equilibrium was computed for the expected genotype distribution using the standard goodness-of-fit test. Multivariate analysis of covariance (MANCOVA) was used to compare WMS-III subscales between two groups; age, educational level, and gender were covariates. Multivariate linear regression analyses were done to explore the interaction between BDNF variants and plasma concentrations of BDNF on memory subscales. Although the Bonferroni correction is the popularly used correction for multiple comparisons, it is too conservative and leads to type II error. Thus, we used the false discovery rate method suggested by Benjamini and Hochberg (1995) to correct for multiple correlations.

## Results

### Demographic Data and Clinical Characteristics

We enrolled 363 participants (228 BD-II and 135 HC). BD-II group was significantly older (*F* = 7.59, *p* = 0.006), had significantly more females (*F* = 4.66, *p* = 0.03), and had significantly less formal education (*F* = 45.07, *p* < 0.005) than did the HC group (Table 1). Plasma concentrations of BDNF were significantly higher in the HC group than in the BD-II group (*F* = 3.94, *p* = 0.04).

**Table 1:** Demographic characteristics and *BDNF* allele and genotype distributions in healthy controls and patients with BP-II.

Variables	Bipolar II disorder (*n* = 228)	Healthy controls (*n* = 135)	F/χ^2^ (*p*-value)
Gender (Male/Female)	105/123	78/57	4.66 (0.03)
Age (Mean ± SD)	35.17 ± 13.26	31.69 ± 7.94	7.59 (0.006)
Educational level (years)	13.27 ± 3.28	15.31 ± 1.65	44.52 (<0.005)
Age at onset of disorder	14.75 ± 4.43	–	
Duration of illness (years)	19.63 ± 12.57	–	
Hamilton depression rating scale score	15.82 ± 4.11	–	
Young mania rating scale score	12.81 ± 3.45	–	
Plasma concentrations of BDNF (ng/ml)	13.86 ± 13.04	16.53 ± 9.15	3.94 (0.048)
BDNF genotype (n, %)			7.81 (0.02)
*Met/Met*	66 (28.9%)	33 (24.4%)	
*Val/Met*	123 (53.9%)	62 (45.9%)	
*Val/Val*	39 (17.1%)	40 (29.6%)	
BDNF allele frequency (n, %)			4.93 (0.03)
*Met*	255 (55.9%)	128 (47.4%)	
*Val*	201 (45.1%)	142 (52.6%)	
**WECHSLER MEMORY SCALE-THIRD EDITION (WMS-III)**			
Auditory immediate (AIM)	100.73 ± 43.20	106.84 ± 13.44	2.31 (0.13)
Visual immediate (VIM)	95.95 ± 17.46	100.26 ± 14.50	4.95 (0.03)
Immediate memory (IM)	96.57 ± 17.26	104.35 ± 12.22	18.62 (<0.0005)
Auditory delayed (ADM)	97.83 ± 18.47	106.98 ± 13.00	25.17 (<0.0005)
Visual delayed (VDM)	95.53 ± 18.31	98.96 ± 13.95	3.53 (0.06)
Auditory recognition delayed (ARDM)	97.10 ± 17.15	107.15 ± 13.01	34.00 (<0.0005)
General memory (GM)	96.37 ± 17.91	104.36 ± 13.08	20.07 (<0.0005)
Working memory (WM)	95.07 ± 14.69	104.53 ± 15.06	34.30 (<0.0005)


To explore which clinical characteristics and demographic variables are associated with plasma concentrations of BDNF, demographic data, age and gender, clinical characteristics, HDRS score, YMRS score, age at onset of BD-II, and duration of illness were independent variables in the multivariate linear regression analyses, and plasma concentrations of BDNF were dependent variables. Stepwise linear regression showed a significant association between plasma concentrations of BDNF level and duration of illness and HDRS scores (*F* = 4.55, *p* = 0.01). Stepwise linear regression also showed that the plasma concentrations of BDNF were significantly negatively associated with the duration of illness (β = -0.17, *t* = -2.51, *p* = 0.017) and positively associated with HDRS scores (β = 0.14, *t* = 2.00, *p* = 0.047).

Memory was assessed using a multivariate analysis of covariance (MANCOVA) with WMSI-III subscales as dependent variables, group (HC and BD-II) as independent variables, and age, educational level, and gender as covariates. There was no significant interaction between the variables of group, age, gender, and educational level (*ps* > 0.05). A subsequent multivariate analysis of variance (MANOVA) that compared the subscales of the WMS-III between groups showed a significant difference between the groups (*F* = 7.50, *p* < 0.0005). In addition, the BD-II patients had significantly lower scores on five subtests of eight indices: WMS-IM, WMS-ADM, WMS-ARDM, WMS-GM, and WMS-WM after the recommended *p*-value correction of multiple comparisons by Benjamini and Hochberg (1995) (Table 1).

### Genotype Effects on Memory Subscales Between Patients and Healthy Controls

The distribution of *BDNF Val^66^Met* genotypes for all participants in the study was as follows: 79 *Val/Val*; 185 *Val/Met*; and 99 *Met/Met*. Allele frequencies were 47.2% for *Val* and 52.8% for *Met*. Genotypes were in Hardy-Weinberg equilibrium using the standard goodness-of-fit test: χ^2^ = 2.34, *p* > 0.05. Genotypes in the BD-II and the HC groups were in Hardy-Weinberg equilibrium, according to the standard goodness-of-fit test: χ^2^ = 2.12, *p* > 0.05 and χ^2^ = 0.845, *p* > 0.05, respectively.

BDNF *Val^66^Met* frequencies were significantly different between the two groups (χ^2^ = 7.81, *p* = 0.02). The frequency of *Val/Val* genotype carriers (17.1%) was significantly lower than the frequency of *Met/Met* genotype carriers (28.9%) in the BD-II group, but not in the HC (29.6% *Val*-homozygote carriers vs. 24.4% *Met*-homozygote carriers) (Table 1).

To explore the effect and association between *BDNF Val^66^Met* genotypes and plasma concentration of BDNF on WMS-III subscales between two groups, stepwise multivariate linear regression analyses were done. In the following analyses, the demographic variables, age, educational level and gender, and BDNF variant and plasma concentration BDNF were counted as independent variables on each subscale of the WMS-III. There was a significant effect of BDNF variants and educational level on WMS-ADM (*F* = 5.19, *p* = 0.006), but not on other memory subscales (Table 2). For other subscales of the WMS-III, educational level and gender seemed to have a significant effect on all variables for both groups. However, the effect of BDNF polymorphisms was found only in the BD-II group, and for some memory indices.

**Table 2:** Linear regression analyses with stepwise for each subscale of WMS-III in the BP-II group.

	Educational level (β, *t*, *p*)	Gender (β, *t*, *p*)	BDNF variant (β, *t*, *p*)	F (*p*-value*)*
WMS-AIM	β = 0.17, *t* = 2.48 (0.01)	–	–	5.90 (0.02)
WMS-VIM	β = 0.18, *t* = 2.69 (0.008)	β = 0.31, *t* = 4.69 (<0.0005)	–	12.49 (<0.0005)
WMS-IM	β = 0.22, *t* = 3.39 (0.001)	β = 0.25, *t* = 3.83 (<0.0005)	–	10.79 (<0.0005)
WMS-ADM	β = 0.22, *t* = 3.28 (0.001)	β = 0.20, *t* = 3.00 (0.003)	β = 0.17, *t* = 2.52 (0.01)	6.79 (<0.0005)
WMS-VDM	–	β = 0.26, *t* = 4.03 (<0.0005)	–	16.23 (<0.0005)
WMS-ADRM	β = 0.24, *t* = 3.48 (0.001)	β = 0.18, *t* = 2.70 (0.007)	β = 0.14, *t* = 2.18 (0.03)	6.29 (<0.0005)
WMS-GM	β = 0.21, *t* = 3.16 (0.002)	β = 0.26, *t* = 3.98 (<0.0005)	β = 0.13, *t* = 2.03 (0.04)	7.84 (<0.0005)
WMS-WM	β = 0.31, *t* = 4.71 (<0.0005)	–	–	22.22 (<0.0005)


*WMS-AIM, auditory immediate memory index; WMS-VIM, visual immediate memory index; WMS-IM, immediate memory index; WMS-ADM, auditory delayed memory index; WMS-ADRM, auditory delayed recall memory index; WMS-GM, general memory index; WMS-WM, working memory index.*

Subsequently, taking the number of *Val* alleles into account, linear regression analyses in BD-II patients with the *Val* homozygote variant showed a significantly negative association with the WMS-ADM (β = -4.24, *t* = -2.36, *p* = 0.02). Moreover, a comparison of each memory subscale of the WMS-III stratified by different BDNF variants in both groups showed no significant effect of BDNF polymorphisms in the HC group. However, BD-II patients with the *Val* homozygote had significantly higher visual immediate memory scores after correction for multiple comparisons (Table 3). Furthermore, a comparison of the plasma concentrations of BDNF between the two groups stratified by BDNF variants showed that among Met homozygote, the BD-II patients with the Met homozygote had significantly lower plasma concentrations of BDNF than did the HCs. There was no significant interaction between BDNF polymorphisms and plasma BDNF levels (*F* = 2.32, *p* = 0.1) in either group, which indicated that the BDNF Val^66^Met polymorphism and the plasma concentration of BDNF might be independent of each other in both groups.

**Table 3:** Comparisons of scores on the WMS-III subtests by genotype groupings in the BP and HC groups.

	Bipolar II disorder			F (*p)*	Healthy Controls			F (*p)*
	*Met/Met*	*Val/Met*	*Val/Val*		*Met/Met*	*Val/Met*	*Val/Val*	
**WECHSLER MEMORY SCALE-THIRD EDITION (WMS-III)**					
Auditory immediate (AIM)	97.86 ± 15.16	101.68 ± 57.20	102.59 ± 15.25	0.21 (0.81)	108.10 ± 13.05	106.08 ± 12.18	106.98 ± 15.70	0.24 (0.786)
Visual immediate (VIM)	97.24 ± 16.25	93.02 ± 18.72	103.00 ± 12.71	5.28 (0.006)^∗^	99.79 ± 19.92	101.11 ± 12.08	99.33 ± 12.90	0.20 (0.815)
Immediate memory (IM)	97.33 ± 15.90	94.36 ± 18.41	103.28 ± 14.00	4.12 (0.017)	106.30 ± 14.02	104.10 ± 10.95	103.13 ± 12.61	0.63 (0.533)
Auditory delayed (ADM)	96.39 ± 18.00	96.17 ± 18.67	105.44 ± 17.08	4.11 (0.018)	106.73 ± 13.08	105.98 ± 11.98	108.73 ± 14.52	0.55 (0.581)
Visual delayed (VDM)	97.24 ± 18.59	92.58 ± 18.87	101.79 ± 13.97	4.27 (0.015)	97.12 ± 15.10	100.52 ± 13.88	98.05 ± 13.12	0.76 (0.472)
Auditory recognition delayed (ARDM)	95.68 ± 16.05	96.28 ± 18.35	102.05 ± 14.36	2.01 (0.137)	108.18 ± 14.51	106.05 ± 12.32	108.00 ± 12.95	0.41 (0.666)
General memory (GM)	96.32 ± 17.16	94.10 ± 18.95	103.49 ± 13.94	4.16 (0.017)	103.15 ± 15.16	104.32 ± 12.25	105.40 ± 12.73	0.27 (0.768)
Working memory (WM)	96.12 ± 13.12	94.33 ± 15.87	95.59 ± 13.59	0.35 (0.708)	99.45 ± 20.29	107.44 ± 11.97	104.20 ± 13.49	3.13 (0.047)
Plasma concentration of BDNF (ng/ml)	11.26 ± 5.17	14.11 ± 7.33	12.98 ± 7.93	2.12 (0.122)	17.42 ± 8.81	15.27 ± 9.03	17.73 ± 9.56	1.09 (0.034)


*There was a linear positive correlation between the number of Val alleles and the ARDM in BP-II (p < 0.05); ^∗^significant level after p-value correction according to FDR.*

## Discussion

Our findings were that BD-II patients had less formal education than did the HCs, which indicates that BD interrupted the patients’ education (Breslau et al., 2008). The BDNF genotype did not affect the age of onset of BD, which was consistent with Hong et al. (2003). BD-II patients with the *Val/Val* variant had significantly better memory scores on the Auditory Delayed Memory index, Auditory Delayed Recognition Memory index, and General Memory index among patients with BDNF genotypes. This finding supported a claim (Egan et al., 2003) that the *Val^66^Met* polymorphism is associated with hippocampal BDNF production and hippocampal structural and functional changes.

Compared with other factors, the HDRS score was more likely to predict the plasma concentrations of BDNF in BD-II patients, which was consistent with Brunoni et al. (2008), who reported an association between BDNF levels and BD-II patients with chronic depression. The neuroplastic changes in BD-II might be caused by depression instead of hypomania. Larger samples are required to confirm this association between peripheral levels (both plasma and serum) of BDNF and mood episodes of BD-II. Other studies have reported that serum BDNF levels rise during manic and depressive episodes, but because our study was not longitudinal, we were unable to confirm this claim.

We found no significant association between BDNF variants and plasma concentrations of BDNF in our study, which was consistent with Tramontina et al. (2007), who reported in euthymic Caucasians. They concluded that the BDNF polymorphism might play a role during acute episodes, as suggested by Cunha et al. (2006). Previous study reported significantly different frequencies for BDNF *Val^66^Met* genotypes and alleles between Asian (Korea) and Caucasian (Croatia) (Pivac et al., 2009). They found that significantly higher frequency of *Met* allele (46.3%) in Asian country compared to 53.7% in the Caucasian. In Shen et al. (2018)’s review, that approximate 50% of Asians carried *Val*/*Met* genotype, but in the Caucasians, more than half carried *Val*/*Val* genotype. The results in our study was consistent with previous finding. The *BDNF* *Met* allele may have a specific role in memory dysfunction among BD-II patients. Unlike the HCs, patients with BD-II the *Met-*homozygote scored consistently lower than did their *Val*-homozygous counterparts on the WMS-ADM subscale and had lower plasma levels of BDNF. We did not find a superior performance of *Val*-homozygote carriers to the other two genotype groups on the WMS-ADM in the HC, which was previously reported (Chang and Yeh, 2012; Yeh et al., 2012) in undergraduate students. Another possibility is that the undergraduate students were not screened for their history of mental illness. The mechanisms through which the *BDNF Val^66^Met* variant affects memory warrants further investigation. The negative association between BDNF level and duration of BD-II indicates that long-term BD-II reduces BDNF levels more than does short-term BD-II (Barbosa et al., 2013). Our findings were consistent with Pacheco et al. (2012), who reported an association between BDNF genotype and memory in older adults which implied that BD-II is a neurodegenerative disorder. However, this hypothesis needs further investigation.

Our study has several limitations that prevent the results from being generalized. First, the sample size is small. Secondly, this cross-sectional study does not allow examination of the interaction between BDNF plasma concentrations and memory: a longitudinal study is necessary. Third, all the participants were Han Chinese; thus, generalizing our findings to other ethnic groups is probably not possible. Using a transmission disequilibrium test (TDT) might confirm our results. Finally, future studies are needed to clarify how BDNF concentrations might affect memory in BD-II patients.

## Conclusion

The association between the *BDNF Met* allele and poor memory, especially auditory delayed memory in BD-II patients, suggests a specific role of the *BDNF Val^66^Met* variant in some aspects of memory dysfunction. In addition, peripheral BDNF levels might be a biomarker of auditory memory performance in BD-II patients. The association between decreased plasma concentrations of BDNF and the degree of cognitive impairment in BD-II patients appears to be independent of the presence of the *BDNF Val^66^Met* polymorphism.

## Author Contributions

Y-HC wrote the first draft of this manuscript and designed this study with R-BL. T-YW, S-YL, S-LC, and C-CH managed the lab work and statistical analyses. T-YW, C-CH, PSC, YKY, J-SH, and R-BL managed the patients’ recruitment and literature review. This study was reviewed by all authors.

## Conflict of Interest Statement

The authors declare that the research was conducted in the absence of any commercial or financial relationships that could be construed as a potential conflict of interest.

## References

[B1] Altar C. A. Cai N. Bliven T. Juhasz M. Conner J. M. Acheson A. L. (1997). Anterograde transport of brain-derived neurotrophic factor and its role in the brain. *Nature* 389 856–860. 10.1038/39885 9349818

[B2] Barbosa I. G. Rocha N. P. Miranda A. S. Huguet R. B. Bauer M. E. Reis H. J. (2013). Increased BDNF levels in long-term bipolar disorder patients. *Rev. Bras Psiquiatr.* 35 67–69. 10.1016/j.rbp.2012.05.011 23567603

[B3] Bearden C. E. Glahn D. C. Monkul E. S. Barrett J. Najt P. Villarreal V. (2006). Patterns of memory impairment in bipolar disorder and unipolar major depression. *Psychiatry Res.* 142 139–150. 10.1016/j.psychres.2005.08.010 16631256

[B4] Bearden C. E. Hoffman K. M. Cannon T. D. (2001). The neuropsychology and neuroanatomy of bipolar affective disorder: a critical review. *Bipolar Disord.* 3 106–150; discussion 151–103. 10.1034/j.1399-5618.2001.030302.x 11465675

[B5] Benjamini Y. Hochberg Y. (1995). Controlling the false discovery rate: a practical and powerful approach to multiple testing. *J. R. Stat. Soc. Ser. B* 57 289–300.

[B6] Berk M. Kapczinski F. Andreazza A. C. Dean O. M. Giorlando F. Maes M. (2011). Pathways underlying neuroprogression in bipolar disorder: focus on inflammation, oxidative stress and neurotrophic factors. *Neurosci. Biobehav. Rev.* 35 804–817. 10.1016/j.neubiorev.2010.10.001 20934453

[B7] Breslau J. Lane M. Sampson N. Kessler R. C. (2008). Mental disorders and subsequent educational attainment in a US national sample. *J. Psychiatr. Res.* 42 708–716. 10.1016/j.jpsychires.2008.01.016 18331741 PMC2748981

[B8] Brunoni A. R. Lopes M. Fregni F. (2008). A systematic review and meta-analysis of clinical studies on major depression and BDNF levels: implications for the role of neuroplasticity in depression. *Int. J. Neuropsychopharmacol.* 11 1169–1180. 10.1017/s1461145708009309 18752720

[B9] Cavanagh J. T. Van Beck M. Muir W. Blackwood D. H. (2002). Case-control study of neurocognitive function in euthymic patients with bipolar disorder: an association with mania. *Br. J. Psychiatry* 180 320–326. 10.1192/bjp.180.4.320 11925354

[B10] Chang C.-Y. Yeh T.-K. (2012). “From gene to educaiton-the ECNG research framework: educaiton, cognition, neuroscience, and gene,” in *Biology Education for Social and Sustainable Development*, eds Kim M. Diong C. H. (Dordrecht: Sense Publishers).

[B11] Charney A. W. Ruderfer D. M. Stahl E. A. Moran J. L. Chambert K. Belliveau R. A. (2017). Evidence for genetic heterogeneity between clinical subtypes of bipolar disorder. *Transl. Psychiatry* 7:e993. 10.1038/tp.2016.242 28072414 PMC5545718

[B12] Chen S. L. Lee S. Y. Chang Y. H. Chen S. H. Chu C. H. Wang T. Y. (2014). The BDNF Val66Met polymorphism and plasma brain-derived neurotrophic factor levels in Han Chinese patients with bipolar disorder and schizophrenia. *Prog. Neuropsychopharmacol. Biol. Psychiatry* 51 99–104. 10.1016/j.pnpbp.2014.01.012 24468644 PMC7137229

[B13] Ciammola A. Sassone J. Cannella M. Calza S. Poletti B. Frati L. (2007). Low brain-derived neurotrophic factor (BDNF) levels in serum of Huntington’s disease patients. *Am. J. Med. Genet. B Neuropsychiatr. Genet.* 144B, 574–577. 10.1002/ajmg.b.30501 17427191

[B14] Cunha A. B. Frey B. N. Andreazza A. C. Goi J. D. Rosa A. R. Goncalves C. A. (2006). Serum brain-derived neurotrophic factor is decreased in bipolar disorder during depressive and manic episodes. *Neurosci. Lett.* 398 215–219. 10.1016/j.neulet.2005.12.085 16480819

[B15] Dickerson F. Boronow J. J. Stallings C. Origoni A. E. Cole S. K. Yolken R. H. (2004). Cognitive functioning in schizophrenia and bipolar disorder: comparison of performance on the repeatable battery for the assessment of neuropsychological status. *Psychiatry Res.* 129 45–53. 10.1016/j.psychres.2004.07.002 15572184

[B16] Dickerson F. B. Boronow J. J. Stallings C. R. Origoni A. E. Cole S. Yolken R. H. (2004). Association between cognitive functioning and employment status of persons with bipolar disorder. *Psychiatr. Serv.* 55 54–58. 10.1176/appi.ps.55.1.54 14699201

[B17] Egan M. F. Kojima M. Callicott J. H. Goldberg T. E. Kolachana B. S. Bertolino A. (2003). The BDNF val66met polymorphism affects activity-dependent secretion of BDNF and human memory and hippocampal function. *Cell* 112 257–269. 12553913 10.1016/s0092-8674(03)00035-7

[B18] Endicott J. Spitzer R. L. (1978). A diagnostic interview: the schedule for affective disorders and schizophrenia. *Arch. Gen. Psychiatry* 35 837–844. 10.1001/archpsyc.1978.01770310043002 678037

[B19] Fernandes B. S. Molendijk M. L. Köhler C. A. Soares J. C. Leite C. M. G. S. Machado-Vieira R. (2015). Peripheral brain-derived neurotrophic factor (BDNF) as a biomarker in bipolar disorder: a meta-analysis of 52 studies. *BMC Med.* 13:289. 10.1186/s12916-015-0529-7 26621529 PMC4666054

[B20] Gualtieri C. T. Johnson L. G. (2006). Comparative neurocognitive effects of 5 psychotropic anticonvulsants and lithium. *MedGenMed*8:46. 17406176 PMC1781293

[B21] Gunstad J. Benitez A. Smith J. Glickman E. Spitznagel M. B. Alexander T. (2008). Serum brain-derived neurotrophic factor is associated with cognitive function in healthy older adults. *J. Geriatr. Psychiatry Neurol.* 21 166–170. 10.1177/0891988708316860 18503034

[B22] Guzowski J. F. Lyford G. L. Stevenson G. D. Houston F. P. McGaugh J. L. Worley P. F. (2000). Inhibition of activity-dependent arc protein expression in the rat hippocampus impairs the maintenance of long-term potentiation and the consolidation of long-term memory. *J. Neurosci.* 20 3993–4001. 10.1523/JNEUROSCI.20-11-03993.2000 10818134 PMC6772617

[B23] Hamilton M. (1960). A rating scale for depression. *J. Neurol. Neurosurg. Psychiatry* 23 56–62. 10.1136/jnnp.23.1.56 14399272 PMC495331

[B24] Hamilton M. (1967). Development of a rating scale for primary depressive illness. *Br. J. Soc. Clin. Psychol.* 6 278–296. 10.1111/j.2044-8260.1967.tb00530.x 6080235

[B25] Hariri A. R. Goldberg T. E. Mattay V. S. Kolachana B. S. Callicott J. H. Egan M. F. (2003). Brain-derived neurotrophic factor val66met polymorphism affects human memory-related hippocampal activity and predicts memory performance. *J. Neurosci.* 23 6690–6694. 10.1523/JNEUROSCI.23-17-06690.2003 12890761 PMC6740735

[B26] Hong C. J. Huo S. J. Yen F. C. Tung C. L. Pan G. M. Tsai S. J. (2003). Association study of a brain-derived neurotrophic-factor genetic polymorphism and mood disorders, age of onset and suicidal behavior. *Neuropsychobiology* 48 186–189. 10.1159/000074636 14673216

[B27] Huang S. Y. Lin W. W. Ko H. C. Lee J. F. Wang T. J. Chou Y. H. (2004). Possible interaction of alcohol dehydrogenase and aldehyde dehydrogenase genes with the dopamine D2 receptor gene in anxiety-depressive alcohol dependence. *Alcohol. Clin. Exp. Res.* 28 374–384. 10.1097/01.ALC.0000117832.62901.61 15084894

[B28] Jamrozinski K. Gruber O. Kemmer C. Falkai P. Scherk H. (2009). Neurocognitive functions in euthymic bipolar patients. *Acta Psychiatr. Scand.* 119 365–374. 10.1111/j.1600-0447.2008.01320.x 19076115

[B29] Judd L. L. Akiskal H. S. Schettler P. J. Coryell W. Maser J. Rice J. A. (2003). The comparative clinical phenotype and long term longitudinal episode course of bipolar I and II: a clinical spectrum or distinct disorders? *J. Affect. Disord.* 73 19–32. 10.1016/S0165-0327(02)00324-5 12507734

[B30] Kapczinski F. Frey B. N. Kauer-Sant’Anna M. Grassi-Oliveira R. (2008a). Brain-derived neurotrophic factor and neuroplasticity in bipolar disorder. *Expert Rev. Neurother.* 8 1101–1113. 10.1586/14737175.8.7.1101 18590480

[B31] Kapczinski F. Vieta E. Andreazza A. C. Frey B. N. Gomes F. A. Tramontina J. (2008b). Allostatic load in bipolar disorder: implications for pathophysiology and treatment. *Neurosci. Biobehav. Rev.* 32 675–692. 10.1016/j.neubiorev.2007.10.005 18199480

[B32] Kauer-Sant’Anna M. Kapczinski F. Andreazza A. C. Bond D. J. Lam R. W. Young L. T. (2009). Brain-derived neurotrophic factor and inflammatory markers in patients with early- vs. late-stage bipolar disorder. *Int. J. Neuropsychopharmacol.* 12 447–458. 10.1017/S1461145708009310 18771602

[B33] Kim H. W. Rapoport S. I. Rao J. S. (2010). Altered expression of apoptotic factors and synaptic markers in postmortem brain from bipolar disorder patients. *Neurobiol. Dis.* 37 596–603. 10.1016/j.nbd.2009.11.010 19945534 PMC2823851

[B34] Levy B. Stephansky M. R. Dobie K. C. Monzani B. A. Medina A. M. Weiss R. D. (2009). The duration of inpatient admission predicts cognitive functioning at discharge in patients with bipolar disorder. *Compr. Psychiatry* 50 322–326. 10.1016/j.comppsych.2008.09.005 19486730 PMC3146314

[B35] Lin P. Y. (2009). State-dependent decrease in levels of brain-derived neurotrophic factor in bipolar disorder: a meta-analytic study. *Neurosci. Lett.* 466 139–143. 10.1016/j.neulet.2009.09.044 19786073

[B36] Lu Y. Christian K. Lu B. (2008). BDNF: a key regulator for protein synthesis-dependent LTP and long-term memory? *Neurobiol. Learn. Mem.* 89 312–323. 10.1016/j.nlm.2007.08.018 17942328 PMC2387254

[B37] Mandolini G. M. Lazzaretti M. Pigoni A. Delvecchio G. Soares J. C. Brambilla P. (2018). The impact of BDNF Val66Met polymorphism on cognition in bipolar disorder: a review. *J. Affect. Disord.* 243 552–558. 10.1016/j.jad.2018.07.054 30078664

[B38] Martinez-Aran A. Vieta E. Reinares M. Colom F. Torrent C. Sanchez-Moreno J. (2004). Cognitive function across manic or hypomanic, depressed, and euthymic states in bipolar disorder. *Am. J. Psychiatry* 161 262–270. 10.1176/appi.ajp.161.2.262 14754775

[B39] Murphy F. C. Sahakian B. J. (2001). Neuropsychology of bipolar disorder. *Br. J. Psychiatry Suppl.* 41 s120–s127. 10.1192/bjp.178.41.s120 11450171

[B40] Neves-Pereira M. Mundo E. Muglia P. King N. Macciardi F. Kennedy J. L. (2002). The brain-derived neurotrophic factor gene confers susceptibility to bipolar disorder: evidence from a family-based association study. *Am. J. Hum. Genet.* 71 651–655. 10.1086/342288 12161822 PMC379201

[B41] Pacheco J. Beevers C. G. McGeary J. E. Schnyer D. M. (2012). Memory monitoring performance and PFC activity are associated with 5-HTTLPR genotype in older adults. *Neuropsychologia* 50 2257–2270. 10.1016/j.neuropsychologia.2012.05.030 22705442 PMC3400137

[B42] Pan W. Banks W. A. Fasold M. B. Bluth J. Kastin A. J. (1998). Transport of brain-derived neurotrophic factor across the blood-brain barrier. *Neuropharmacology* 37 1553–1561. 10.1016/S0028-3908(98)00141-5 9886678

[B43] Pivac N. Kim B. Nedic G. Joo Y. H. Kozaric-Kovacic D. Hong J. P. (2009). Ethnic differences in brain-derived neurotrophic factor Val66Met polymorphism in Croatian and Korean healthy participants. *Croat. Med. J.* 50 43–48. 10.3325/cmj.2009.50.43 19260143 PMC2657559

[B44] Poo M. M. (2001). Neurotrophins as synaptic modulators. *Nat. Rev. Neurosci.* 2 24–32. 10.1038/35049004 11253356

[B45] Quraishi S. Frangou S. (2002). Neuropsychology of bipolar disorder: a review. *J. Affect. Disord.* 72 209–226. 10.1016/S0165-0327(02)00091-5 12450638

[B46] Rihmer Z. Kiss K. (2002). Bipolar disorders and suicidal behaviour. *Bipolar Disord.* 4(Suppl. 1), 21–25. 10.1034/j.1399-5618.4.s1.3.x 12479671

[B47] Shen T. You Y. Joseph C. Mirzaei M. Klistorner A. Graham S. L. (2018). BDNF polymorphism: a review of its diagnostic and clinical relevance in neurodegenerative disorders. *Aging Dis.* 9 523–536. 10.14336/AD.2017.0717 29896439 PMC5988606

[B48] Simonsen C. Sundet K. Vaskinn A. Birkenaes A. B. Engh J. A. Hansen C. F. (2008). Neurocognitive profiles in bipolar I and bipolar II disorder: differences in pattern and magnitude of dysfunction. *Bipolar Disord.* 10 245–255. 10.1111/j.1399-5618.2007.00492.x 18271903

[B49] Toh Y. L. Ng T. Tan M. Tan A. Chan A. (2018). Impact of brain-derived neurotrophic factor genetic polymorphism on cognition: a systematic review. *Brain Behav.* 8:e01009. 10.1002/brb3.1009 29858545 PMC6043712

[B50] Tramontina J. Frey B. N. Andreazza A. C. Zandona M. Santin A. Kapczinski F. (2007). Val66met polymorphism and serum brain-derived neurotrophic factor levels in bipolar disorder. *Mol. Psychiatry* 12 230–231. 10.1038/sj.mp.4001941 17325716

[B51] Vieta E. Gasto C. Otero A. Nieto E. Vallejo J. (1997). Differential features between bipolar I and bipolar II disorder. *Compr. Psychiatry* 38 98–101. 10.1016/S0010-440X(97)90088-2 9056128

[B52] Vieta E. Suppes T. (2008). Bipolar II disorder: arguments for and against a distinct diagnostic entity. *Bipolar Disord.* 10(1 Pt 2), 163–178. 10.1111/j.1399-5618.2007.00561.x 18199235

[B53] Wang Z. Li Z. Chen J. Huang J. Yuan C. Hong W. (2012). Association of BDNF gene polymorphism with bipolar disorders in Han Chinese population. *Genes Brain Behav.* 11 524–528. 10.1111/j.1601-183X.2012.00797.x 22548711

[B54] Wechsler D. Stone C. (1997). *Wechsler Memory Scale*, 3rd Edn San Antonio, TX: The Psychological Corporation.

[B55] Yeh T. K. Hu C. Y. Yeh T. C. Lin P. J. Wu C. H. Lee P. L. (2012). Association of polymorphisms in BDNF, MTHFR, and genes involved in the dopaminergic pathway with memory in a healthy Chinese population. *Brain Cogn.* 80 282–289. 10.1016/j.bandc.2012.06.005 22940753

[B56] Young R. C. Biggs J. T. Ziegler V. E. Meyer D. A. (1978). A rating scale for mania: reliability, validity and sensitivity. *Br. J. Psychiatry* 133 429–435. 10.1192/bjp.133.5.429 728692

[B57] Yu H. Zhang Z. Shi Y. Bai F. Xie C. Qian Y. (2008). Association study of the decreased serum BDNF concentrations in amnestic mild cognitive impairment and the Val66Met polymorphism in Chinese Han. *J. Clin. Psychiatry* 69 1104–1111. 10.4088/JCP.v69n0710 18505307

[B58] Zhang X. Y. Chen D. C. Xiu M. H. Haile C. N. Luo X. Xu K. (2012). Cognitive and serum BDNF correlates of BDNF Val66Met gene polymorphism in patients with schizophrenia and normal controls. *Hum. Genet.* 131 1187–1195. 10.1007/s00439-012-1150-x 22362486 PMC3671849

